# Exploration of the Immunotyping Landscape and Immune Infiltration-Related Prognostic Markers in Ovarian Cancer Patients

**DOI:** 10.3389/fonc.2022.916251

**Published:** 2022-07-08

**Authors:** Na Zhao, Yujuan Xing, Yanfang Hu, Hao Chang

**Affiliations:** ^1^ Department of Gynecology, Dongying People’s Hospital, Dongying, China; ^2^ Department of Cancer Research, Hanyu Biomed Center Beijing, Beijing, China

**Keywords:** immunophenotyping, immune checkpoints, prognosis, ovarian cancer, immune cell infiltration, immunotherapy

## Abstract

**Background:**

Increasing evidence indicates that immune cell infiltration (ICI) affects the prognosis of multiple cancers. This study aims to explore the immunotypes and ICI-related biomarkers in ovarian cancer.

**Methods:**

The ICI levels were quantified with the CIBERSORT and ESTIMATE algorithms. The unsupervised consensus clustering method determined immunotypes based on the ICI profiles. Characteristic genes were identified with the Boruta algorithm. Then, the ICI score, a novel prognostic marker, was generated with the principal component analysis of the characteristic genes. The relationships between the ICI scores and clinical features were revealed. Further, an ICI signature was integrated after the univariate Cox, lasso, and stepwise regression analyses. The accuracy and robustness of the model were tested by three independent cohorts. The roles of the model in the immunophenoscores (IPS), tumor immune dysfunction and exclusion (TIDE) scores, and immunotherapy responses were also explored. Finally, risk genes (GBP1P1, TGFBI, PLA2G2D) and immune cell marker genes (CD11B, NOS2, CD206, CD8A) were tested by qRT-PCR in clinical tissues.

**Results:**

Three immunotypes were identified, and ICI scores were generated based on the 75 characteristic genes. CD8 TCR pathways, chemokine-related pathways, and lymphocyte activation were critical to immunophenotyping. Higher ICI scores contributed to better prognoses. An independent prognostic factor, a three-gene signature, was integrated to calculate patients’ risk scores. Higher TIDE scores, lower ICI scores, lower IPS, lower immunotherapy responses, and worse prognoses were revealed in high-risk patients. Macrophage polarization and CD8 T cell infiltration were indicated to play potentially important roles in the development of ovarian cancer in the clinical validation cohort.

**Conclusions:**

Our study characterized the immunotyping landscape and provided novel immune infiltration-related prognostic markers in ovarian cancer.

## Introduction

The incidence rate of ovarian cancer (OC) ranks thirdly behind cervical cancer and endometrial cancer in gynecologic cancers, while its fatality rate is the highest (1). Due to atypical symptoms, approximately 70% of OC patients are in advanced stages at diagnosis (2). Surgical resection combined with chemotherapy based on platinum is the current first-line treatment for OC (3). Although most OC patients are sensitive to chemotherapy, the high relapse rate limited the prognosis (4). Recently, significant advances have been made in surgical treatment and targeted therapy. Some clinical trials like prospective randomized trial AGO DESKTOP III and randomized phase III trial SGOG SOC-1 have provided evidence that the complete (R0) resection with secondary cytoreductive surgery could improve the overall survival of recurrent OC patients (5, 6). PARP inhibitors, Olaparib and Niraparib, have been approved by the United States Food and Drug Administration for the first-line maintenance treatment according to the status of BRCA mutation and usage of bevacizumab as the first-line chemotherapy (7). PARP inhibitors have significant survival benefits for BRCA-mutant patients with platinum-sensitive recurrent ovarian cancer (8, 9).

However, there are few breakthroughs in immunotherapy, which has been a critical treatment modality in melanoma, lung cancer, kidney cancer, and hematological cancer (10, 11). The phase II trial KEYNOTE-100 indicated that the objective response rate (ORR) of pembrolizumab monotherapy was only 8%, while a higher rate of 17% in patients with PD-L1 combined positive score (CPS) ≥ 10 (12). Another phase II trial NCT02853318 reported that pembrolizumab combined bevacizumab and cyclophosphamide brought up to 95% in disease control rate (DCR) and 40% in ORR (13). In general, immune-based strategies imply promising clinical benefits.

Cancer-infiltrating immune cells are important components of the tumor microenvironment (TME) (14). Distinctive sorts of immune cells play different parts in immune responses. For instance, CD8+ T cells, CD4+ T cells, Activated NK cells, and M1 Macrophages usually act as tumor suppressors. Treg cells and M2 Macrophages are generally regarded as tumor-promoting factors. But for the strong cellular and genetic heterogeneity, there is an urgent demand to explore effective biomarkers and characterizations of TME in OC to develop personalized immunotherapies (15, 16). Based on RNA sequencing profiles of two public datasets, comprehensive algorithms were conducted in this study to deeply learn the landscape of ICI and explore effective ICI-related biomarkers for survival prediction and immunotherapy strategies in OC patients.

## Materials and Methods

### Patients and Ethics Statement

The study was approved by the Ethics Committee of Dongying People’s Hospital and carried out strictly following the Declaration of Helsinki. All patients signed the informed consent forms.

### Datasets and Processing for Bioinformatic Analyses

This study included up to 537 solid tissues from the TCGA-OV (n = 329), ICGC-OV-AU (n = 82), and GSE138866 (n = 126) datasets. All the expression data were generated based on the next-generation sequencing technology. The TCGA-OV dataset was downloaded from the UCSC XENA website (https://xenabrowser.net/datapages/), the GSE138866 dataset was downloaded from the Gene Expression Omnibus (GEO) database (https://www.ncbi.nlm.nih.gov/geo/), and the ICGC-OV-AU dataset was downloaded from the International Cancer Genome Consortium (ICGC) database (https://dcc.icgc.org/). The obtained expression profiles were normalized into Transcripts Per Kilobase Million (TPM), and log_2_(X+1) transformed. Batch effects were then removed by using the “Combat” function in the SVA R package (17). The somatic mutation data for the TCGA-OV cohort based on VarScan2 (18) were obtained from the The cancer genome atlas (TCGA) database. Samples with the overall survival time no more than 30 days were excluded.

### Immunotypes Based on the Immune Cell Infiltration 

To quantify the infiltration levels of different immune cells in OC samples, we applied the CIBERSORT R package (19) with theparameter perm = 1,000 to estimate the LM22 signature of ICI for each sample. Results with *P* < 0.05 were considered credible, and we selected 209 samples for further analysis. Moreover, each sample’s immune and stromal score was calculated using the ESTIMATE R package (20). The ICI profiles determined immunotypes by the k-means algorithm in the ConsensusClusterPlus R package to perform consensus clustering based on Euclidean distance (21, 22). The clustering process was performed 1000 times, with each iteration containing 80% of the samples. Survival analysis among different ICI subtypes was conducted. The correlations among the infiltration levels were calculated with the Pearson correlation test. The infiltration fractions of immune cells and the expression levels of common immune checkpoint genes in different ICI immunotypes were differentially analyzed with the Wilcoxon test.

### Differentially Expressed Genes Among the Immunotypes

To identify the DEGs among ICI immunotypes, we conducted empirical Bayes variance moderation with the Limma R package (23). The cutoff was set to adjusted *P*-value < 0.05 and |log_2_ fold-change| > 0.585. Based on the expression profile of identified DEGs, unsupervised consensus clustering was conducted again with the ConsensusClusterPlus R package. Survival analysis among DEGs clusters was performed with the Survival R package.

### The ICI Score Generated With PCA Dimension Reduction

DEGs were initially classified as positive (A) and negative groups (B) correlated with the DEGs clusters changing using the “cor.test” function in R software. The Boruta R package (24) was applied to screen characteristic genes influencing the consensus clustering. Then, principal component analysis (PCA) dimension reduction (25) was conducted with the prcomp function to generate the linear dimension reduction formula based on the expression of all the characteristic genes from the “rotation” results (the matrix of variable loadings). PCA scores were obtained with the “predict” function in positive and negative correlated groups separately. Finally, the ICI score was defined as the following formula: ∑ PC1_A_ - ∑ PC1_B._ Patients with high and low ICI scores were separated based on the cutoff value generated from the “surv_cutpoint” function in the survminer R package. Kaplan-Meier plotter was performed with the Survival R package. The differences between the high- and low-ICI score groups in clinical features (Age, Survival Status and Stages) were further conducted with the Wilcoxon test. Functional enrichment analysis and protein-protein interaction network were performed with the webtool Metascape (26).

### The Relationship Between the ICI Score and Tumor Mutational Burden

Tumor Mutational Burden (TMB) has proven an effective predictor for immunotherapy (27). Non-synonymous mutations of patients in the TCGA-OV cohort were counted. TMB scores were calculated with the number of variants/the length of exons (38 million). The difference of TMB values between the high- and low-ICI score groups was analyzed with the Wilcoxon test. Then, the high- and low-TMB groups were separated with the cutoff generated from the “surv_cutpoint” function in the survminer R package. The somatic alterations landscapes were calculated and plotted with the maftools R package (28). The patients were further separated into four subgroups in combination with the ICI scores. Kaplan-Meier plotters were additionally conducted.

### Development and Validation of the ICI Signature

A multi-gene ICI signature was explored using expression profiles of characteristic genes related to ICI clustering for the overall survival prediction. In detail, we used the TCGA-OV cohort as the training cohort and the ICGC-OV-AU and GSE138866 cohorts as the independent validation cohorts. The univariate Cox regression analysis was performed to screen candidate genes (*P* < 0.05) in the beginning. Least absolute shrinkage and selection operator (LASSO) and stepwise regression analyses were further conducted and a three-gene signature estimating the risk score of each patient was established. Patients were defined as high-risk and low-risk according to the median risk score in the TCGA-OV cohort. With the same cutoff, patients in the two validation cohorts were grouped too. The Kaplan-Meier plotters and receiver operating characteristic (ROC) curves were applied to test the accuracy of the signature.

### The Predictive Roles of the ICI Signature in Immunotherapy

To compare the differences between high-risk and low-risk groups in immunotherapy, we obtained the immunophenoscores (IPS) (29) of the TCGA-OV cohort from The Cancer Immunome Atlas (TCIA) database (https://tcia.at/home) and calculated the tumor immune dysfunction and exclusion (TIDE) scores with the TIDE webtool (http://tide.dfci.harvard.edu/) (30), and quantified the ICI levels with CIBERSORT. Moreover, immunotherapy responses and survival analysis were also explored in the immunotherapy cohort IMvigor210 (31).

### Integration of the Prognostic Nomogram

The independence of the risk score and clinical features (Age, Stages, Grade) was analyzed with multivariate Cox regression analyses. At last, a nomogram including all the 537 patients was constructed with the “rms” R package to predict the overall survival of OC patients. The performance was tested with the Kaplan-Meier and calibration curves.

### Exploration in Clinical Tissues by qRT-PCR

To explore the clinical relevance of the above ICI risk model and the differences in the expression levels of related risk genes (GBP1P1, TGFBI, PLA2G2D) and immune cell marker genes (CD11B, NOS2, CD206, CD8A), we performed the qRT-PCR testing in 42 clinical tissue samples of ovarian cancer. According to the manufacturer’s instructions, total RNA was extracted using the Total RNA Purification Kit (GeneMark, USA). The relative mRNA expression levels (2^-ΔCT^) were quantified after normalization to GAPDH, and the primers used are shown in **Table 1**. The clinical features in this validation cohort are shown in **Table 2**.

**Table 1:** qRT-PCR primers.

Symbol	Accession no.	Forward (5’-3’)	Reverse (5’-3’)
GBP1P1	NR_003133	CTGAGAAGATGGAGAGCGACA	TAAGCAAGCAGGGTTCTTCCC
TGFBI	NM_000358	CTCATCCCAGACTCAGCCAA	TCAACCGCTCACTTCCAGAG
PLA2G2D	NM_012400	GGAACATCCACTGCTCTGACAA	AACGCAGTCGCTTCTGGTAG
CD11B	NM_001145808	TTCCAAGAGAACGCAAGGGG	TAGTCGCACTGGTAGAGGCT
NOS2	NM_000625	CGTGGAGACGGGAAAGAAGT	GACCCCAGGCAAGATTTGGA
CD206	NM_002438	TCAGATATGCCAGGGCGAAAG	GGACATTTGGGTTCGGGAGT
CD8A	NM_001382698	AGACAGTGGAGCTGAAGTGC	TAGGAGGAAGGTGGGACTGG
GAPDH	NM_001256799	GTCTCCTCTGACTTCAACAGCG	ACCACCCTGTTGCTGTAGCCAA

**Table 2:** Clinical features of the clinical tissues.

	Low-Risk (N=21)	High-Risk (N=21)	Overall (N=42)
Age			
≥60	11 (52.4%)	10 (47.6%)	21 (50.0%)
<60	10 (47.6%)	11 (52.4%)	21 (50.0%)
Stage			
II	12 (57.1%)	7 (33.3%)	19 (45.2%)
III	7 (33.3%)	8 (38.1%)	15 (35.7%)
IV	2 (9.5%)	6 (28.6%)	8 (19.0%)
Grade			
G2	5 (23.8%)	4 (19.0%)	9 (21.4%)
G3	16 (76.2%)	17 (81.0%)	33 (78.6%)

### Statistical Analyses

All the statistical analyses were performed in R 4.0.3. The Kaplan-Meier plotters were executed with the log-rank test. The contrasts among three or more groups were conducted with the Kruskal-Wallis test, and the differences between the two groups were performed with the Wilcoxon test or T test. Pearson analysis calculated the correlation coefficient. *P* < 0.05 was considered to be statistically significant.

## Results

### Immune Cell Infiltration Landscape of OC

**Figure 1:**
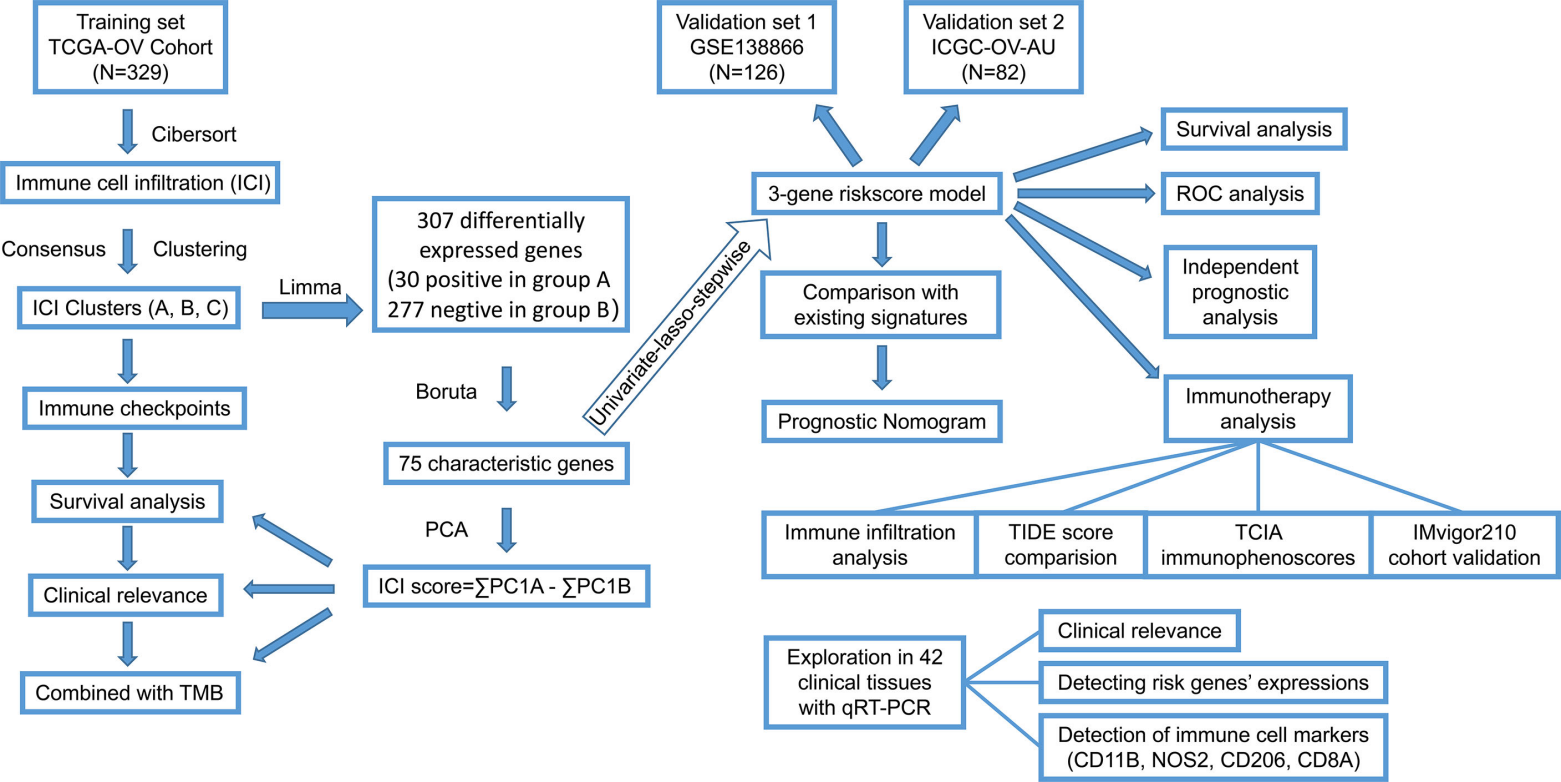
The work flow diagram of this study.

**Figure 2:**
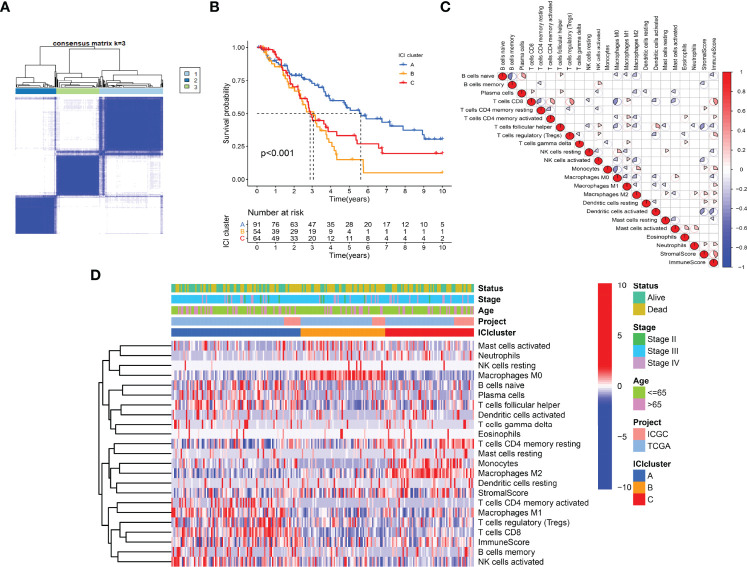
Immunotypes and immune cell infiltration (ICI) landscape of OC. **(A)** ICI subtypes identified with ConsensusClusterPlus. **(B)** Kaplan-Meier curves of ICI subtypes with log-rank test *P* < 0.001. **(C)** Heatmap of Pearson correlation coefficients among immune cells. **(D)** Characteristics of ICI levels and clinical features in ICI clusters.

**Figure 3:**
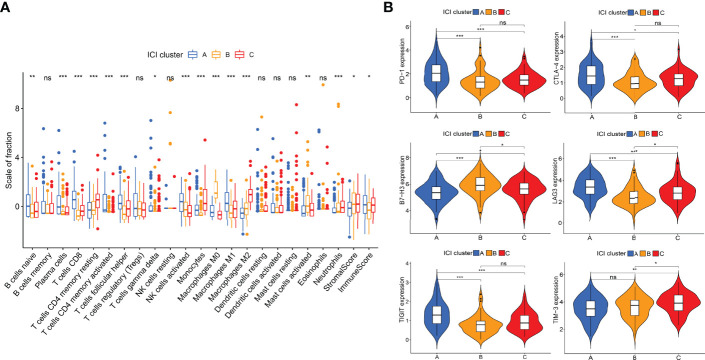
Differential analyses of ICI levels and immune checkpoint genes among ICI clusters. **(A)** Box diagram of ICI levels. Kruskal-Wallis test. **(B)** Violin plots of six immune checkpoint genes. Wilcoxon test. *P < 0.05; **P < 0.01; ***P < 0.001; ns: no significant.

The work flow diagram of this study is constructed (**Figure 1**). Patient characteristics of the three independent cohorts are shown in **Table 3**. The infiltration levels of different immune cells in the TCGA cohort were calculated with CIBERSORT (**Supplementary File S1**), and the immune and stromal scores were calculated with ESTIMATE (**Supplementary File S2**). A total of 209 samples with credible CIBERSORT results were selected. Three ICI subtypes were identified with ConsensusClusterPlus (**Figure 2A**). The overall survival among the three ICI subtypes was significantly different with the log-rank test *P* < 0.001 (**Figure 2B**). ICI cluster A presented the best prognosis while ICI cluster B presented the worst (**Figure 2B**). A heatmap of cellular interaction of the tumor-infiltrating immune cell types was plotted (**Figure 2C**). Significantly positive correlations in ICI levels were found between B cells naïve and plasma cells, B cells memory and NK cells activated, T cells CD8 and T cells CD4 memory, T cells CD8 and T cells regulatory, T cells follicular helper and Dendritic cells activated, NK cell resting and Macrophages M0. While negative correlations were found between B cells naïve and B cells memory, T cells CD8 and Macrophages M0, T cells CD8 and T cells CD4 memory resting, etc (**Figure 2C**). The different characteristics of ICI levels and clinical features in the three ICI clusters were plotted (**Figure 2D**). The differential analysis was conducted with the Kruskal-Wallis test and displayed in a box diagram (**Figure 3A**). B cells naive, Plasma cells, T cells CD8, T cells CD4 memory activated, T cells follicular helper, NK cells activated, and Macrophages M1 were indicated to be increased significantly in ICI cluster A. Macrophages M0 and Mast cells activated were increased significantly in ICI cluster B. Macrophages M2, Monocytes, and T cells CD4 memory resting were increased significantly in ICI cluster C (**Figures 2D** and **3A**). Moreover, the expression levels of ten common immune checkpoint genes were differentially analyzed with the Wilcoxon test. PD-1, CTLA-4, LAG3, and TIGIT were significantly up-regulated in the ICI cluster A. B7-H3 was significantly up-regulated in the ICI cluster B. TIM-3 was significantly up-regulated in the ICI cluster C (**Figure 3B**). However, no significant differences were found for CD47, VISTA, PD-L1, and NKG2A (data not shown).

**Table 3:** Patient characteristics.

	TCGA-OV (N=329)	GSE138866 (N=126)	ICGC-OV-AU (N=82)
Age			
> 65	104 (31.61%)	54 (42.86%)	23 (28.05%)
≤ 65	225 (68.39%)	72 (57.14%)	59 (71.95%)
Stage			
II	19 (5.78%)	2 (1.59%)	0 (0.00%)
III	261 (79.33%)	104 (82.54%)	70 (85.37%)
IV	46 (13.98%)	20 (15.87%)	12 (14.63%)
Unknown	3 (0.01%)	0 (0.00%)	0 (0.00%)
Grade			
G1-G2	38 (11.55%)	0 (0.00%)	NA
G3-G4	283 (86.02%)	126 (100.00%)	NA
Unknown	8 (2.43%)	0 (0.00%)	NA

NA, Not Available

### Differentially Expressed Genes Among the Immunotypes

**Figure 4:**
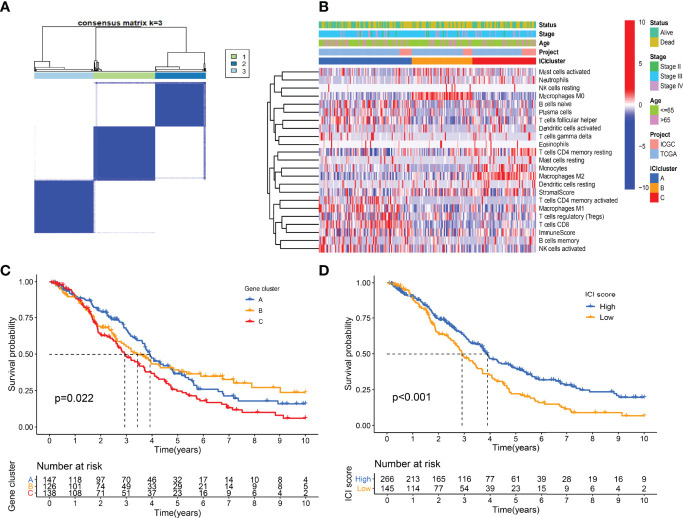
Landscape of differentially expressed gene clusters. **(A)** Unsupervised consensus clustering. **(B)** Characteristics of DEGs expression levels and clinical features in different clusters. **(C)** Kaplan-Meier curve of gene clusters with log-rank test *P* = 0.022. **(D)** Kaplan-Meier curve of gene clusters with log-rank test *P* < 0.001.

A total of 307 differentially expressed genes were identified with the previous threshold. Three differentially expressed gene clusters were generated after unsupervised consensus clustering (**Figure 4A**). The landscape of relationships between clinical features and gene clusters was visualized with the heatmap (**Figure 4B**). Significant survival differences among the three clusters were revealed (*P* = 0.022) (**Figure 4C**).

### The ICI Score Generated With PCA Dimension Reduction

**Figure 5:**
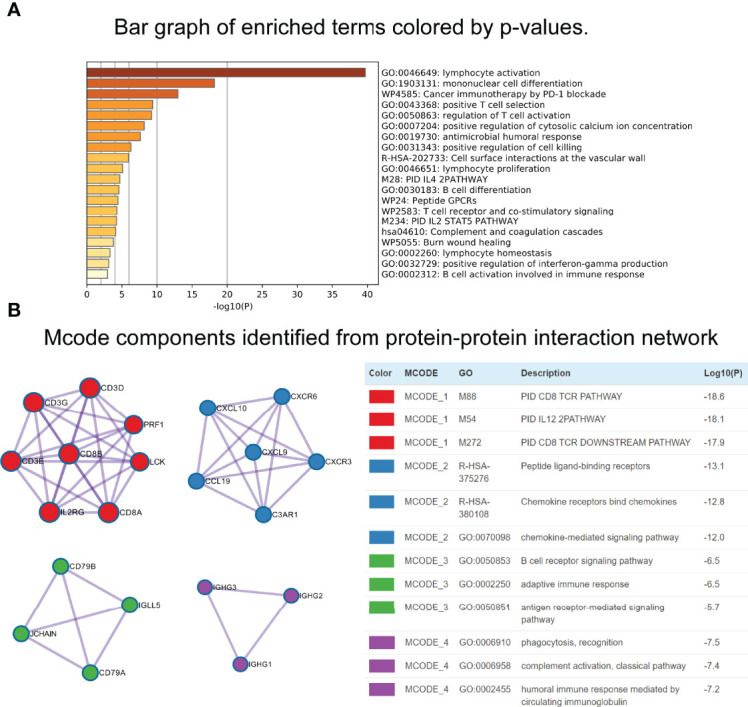
Functional enrichment analyses of characteristic genes. **(A)** Bar graph of enriched terms. **(B)** Mcode components identified from Protein-protein interaction (PPI) network.

**Figure 6:**
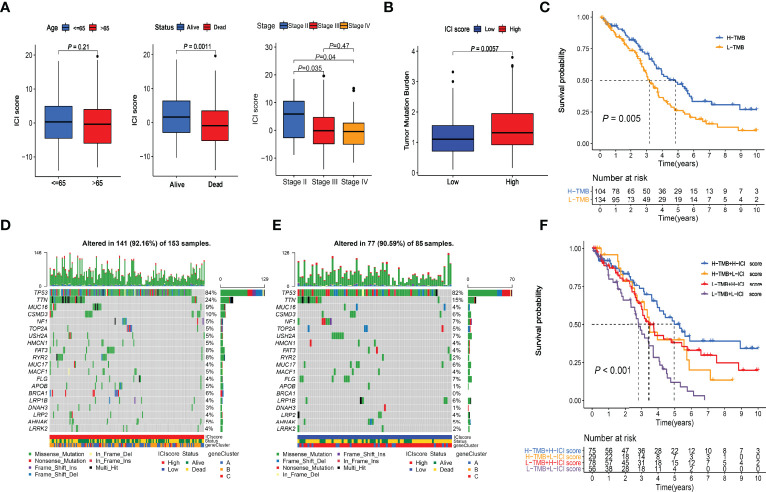
Clinical relevance of ICI score and combined survival analysis with TMB. **(A)** Relationships between ICI scores and Age, Survival Status, and Stages. **(B)** Comparison of TMB values between High and Low ICI score groups. Wilcoxon test *P* = 0.0057. **(C)** Kaplan-Meier curve of High-TMB and Low-TMB groups. Log-rank test *P* = 0.005. **(D)** Somatic alterations landscapes of top 20 genes in high ICI score group. **(E)** Somatic alterations landscapes of top 20 genes in low ICI score group. **(F)** Combined survival analysis with TMB. Log-rank test *P* < 0.001.

Using the “cor.test” function in R software, the 307 DEGs were separated into group A (positive) and group B (negative). With the Boruta R package, there were 75 characteristic genes screened. DEGs and characteristic genes were provided (**Supplementary File S3**). Principal component analysis (PCA) dimension reduction was performed, and PCA scores were obtained in the two groups separately. The ICI scores (**Supplementary File S4**) were generated with the formula ∑ PC1A - ∑ PC1B. The cutoff value of -2.946049 was selected with the “surv_cutpoint” function to define high and low ICI score groups. The Kaplan-Meier plotter indicated that the high ICI group had a significantly (*P* < 0.001) better prognosis than the low ICI group (**Figure 4D**). To better understand the functional differences brought by the 75 characteristic genes, the enrichment analysis (**Figure 5A**) and protein-protein interaction (PPI) network (**Figure 5B**) were performed. Lymphocyte activation, mononuclear cell differentiation, cancer immunotherapy by PD-1 blockades, positive T cell selection, and regulation of T cell activation were the top 5 enriched functions, which showed very high consistency with the ICI landscape. With the Mcode algorithm of Cytoscape software, four components were identified from the PPI network. CD8 TCR pathway, chemokine-mediated signaling pathway, B cell receptor signaling pathway and adaptive immune response were significantly enriched, which might be the main reasons for the immunological heterogeneity. Patients in the Alive group had substantially higher ICI scores than the Dead group. Patients in Stage II had significantly higher ICI scores than Stage III and Stage IV. No difference was found between Age > 65 and group Age ≤ 65, and Stage III and Stage IV (**Figure 6A**).

### The Relationship Between the ICI Score and Tumor Mutational Burden

The TMB scores of the TCGA cohort were calculated (**Supplementary File S5**). Wilcoxon test indicated that the high-ICI group had significantly (*P* = 0.0057) higher TMB values than the low-ICI group (**Figure 6B**). The cutoff value of 1.315789 was selected with the “surv_cutpoint” function, and high- and low-TMB groups were defined. The Kaplan-Meier plotter indicated that the high TMB group had a significantly (*P* = 0.005) better prognosis than the low TMB group (**Figure 6C**), which had good consistency with the survival analysis between the high and low ICI score groups. The somatic alterations landscapes of the top 20 genes in the high ICI score group (**Figure 6D**) and the low ICI score group (**Figure 6E**) were visualized separately. In combination with the ICI scores, patients were further separated into four subgroups. The Kaplan-Meier plotters indicated that the high-TMB & high-ICI group had the best prognosis, while the low-TMB & low-ICI group had the worst. The high-TMB & low-ICI group and the low-TMB & high-ICI group had similar and moderate prognoses (**Figure 6F**).

### Development and Validation of the ICI Signature

**Figure 7:**
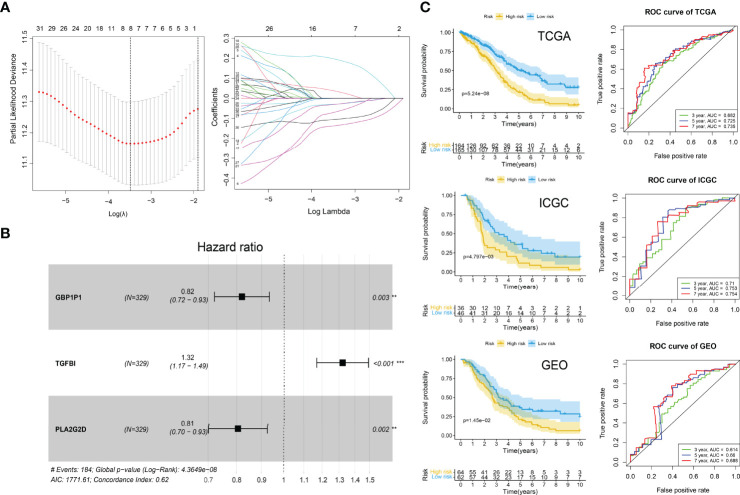
ICI gene signature for survival prediction. **(A)** Lasso regression analysis. Partial likelihood deviance profiles (left). Coefficients profiles (right). **(B)** Hazard ratio of multivariate Cox model. **P < 0.01; ***P < 0.001. **(C)** Kaplan-Meier curves (left) of high-risk and low-risk groups and ROC curves (right) in TCGA, ICGA, and GEO cohorts. Log-rank test.

We used the TCGA-OV cohort as the training cohort and the ICGC-OV-AU and GSE138866 cohorts as the independent validation cohorts. The PCA analyses were performed to confirm the batch effects were acceptable (**Figure S1**). With the 75 characteristic genes screened by Boruta, the univariate Cox regression analysis was conducted in the TCGA-OV cohort first. Thirty-seven genes were identified with *P* < 0.05 (**Supplementary File S6**). After LASSO regression analysis (**Figure 7A**), eight variables (CD3G, IGHG1, MS4A1, IGLC3, GBP1P1, TGFBI, PLA2G2D, EDNRA) were selected for the further stepwise regression analysis. Finally, a three-gene signature was constructed: risk score = (-0.2180 × GBP1P1) + (0.2670 × TGFBI) + (0.2561 × PLA2G2D). All the three genes were significantly (*P* < 0.01) related to the overall survival in the multivariate Cox regression analysis (**Figure 7B**). With the cutoff value of 1.034349, the median risk score of the TCGA-OV cohort, all patients were separated into high and low-risk groups. As expected in the Kaplan-Meier Curves, the high-risk group had a significantly (*P* < 0.01) worse prognosis than the low-risk group in all cohorts (**Figure 7C**). The Area Under Curve (AUC) values of the ROC curves predicting 7-year overall survival were 0.735 in the TCGA-OV cohort, 0.754 in the ICGC-OV-AU cohort, and 0.688 in the GSE138866 cohort (**Figure 6C**).

### The Predictive Roles of the ICI Signature in Immunotherapy

**Figure 8:**
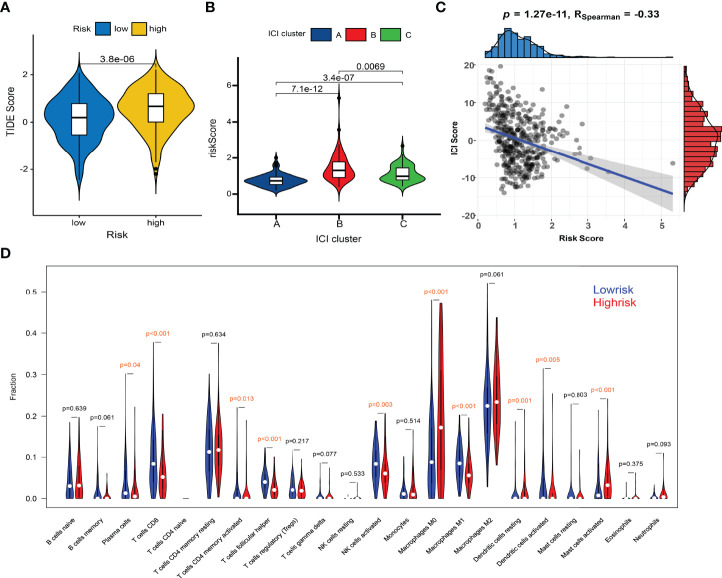
Comparisons of TIDE scores and ICI levels in high-risk and low-risk patients. **(A)** Violin plot of TIDE scores in high- and low-risk groups. **(B)** Risk scores among different immunotypes. **(C)** Spearman correlation between the risk scores and TIDE scores. **(D)** Violin plot of ICI fractions.

**Figure 9:**
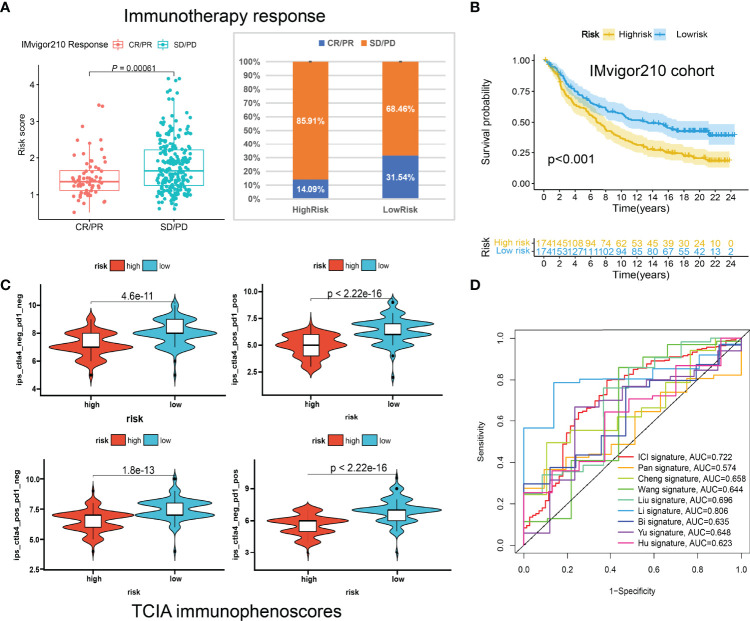
The predictive roles of the ICI signature in immunotherapy. **(A)** Correlations between the risk scores and immunotherapy responses in IMvigor210. **(B)** Kaplan-Meier curve of the high-risk and low-risk patients in IMvigor210. **(C)** The relationship between the risk scores and immunophenoscores (IPS) in the four subgroups based on CTLA4 and PD1 status in TCIA database. **(D)** ROC curves of multiple signatures for the 7-year survival prediction.

Huge differences in responses to immune checkpoint therapy and immune cell infiltrations were found. The TIDE scores in the high-risk group were significantly higher (*P* < 0.0001) (**Figure 8A**), which indicated more serious tumor immune dysfunction and exclusion. Significant differences in risk scores among different immunotypes were discovered as well. Specifically, ICI cluster B had the highest risk scores, ICI cluster A lowest, and ICI cluster C in the middle (**Figure 8B**). There was also a significantly negative correlation between the ICI score and the risk score (*P* < 0.0001, R = -0.33) (**Figure 8C**). The fractions of antitumor immune cells like T cells CD8 (*P* < 0.001), NK cells activated (*P* = 0.003), Macrophages M1 (*P* < 0.001) were significantly higher in low-risk patients (**Figure 8D**). Then, we validated the ICI signature in the immunotherapy cohort IMvigor210, separating patients with a median risk score. Patients in the SD/PD response group had significantly higher risk scores than those in the CR/PR group (**Figure 9A**) (**Supplementary File S7**). Higher response rates were available in the low-risk patients (**Figure 9A**). KM curve also showed the high-risk group had significantly worse prognoses (**Figure 9B**). Further, we obtained the immunophenoscores (IPS) of the TCGA-OV cohort from the TCIA database, which could predict the immunogenicity and immunotherapy response (**Supplementary File S8**). The low-risk group showed significantly higher IPS in all the four subgroups based on CTLA4 and PD1 status (**Figure 9C**), which meant potentially higher immunotherapy response rates.

### Comparisons the ICI Three-Gene Model With Existing Signatures

To further examine the performance of the ICI signature, we retrieved eight published works of literature in the past year and conducted risk scoring with their model variables for all the patients included in our study. The ROC curves for the 7-year survival prediction were plotted with the timeROC R package (**Figure 9D**). The AUC values were 0.722 for our 3-gene ICI signature, 0.574 for the 7-gene Pan signature (32), 0.658 for the 6-gene Cheng signature (33), 0.644 for the 4-gene Wang signature (34), 0.696 for the 8-gene Liu signature (35), 0.806 for the 14-gene Li signature (36), 0.635 for the 8-gene Bi signature (37), 0.648 for the 5-gene Yu signature (38), and 0.623 for the 5-gene Hu signature (39) (**Figure 9D**). Although the AUC value ranks second, less than the 14-gene Wang signature, our ICI signature contains the least number of genes, which means it is more convenient for clinical applications.

### Integration of the ICI-Related Prognostic Nomogram

**Figure 10:**
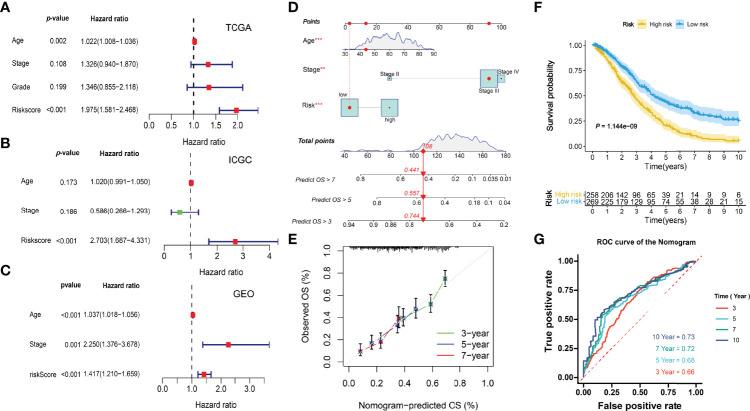
Integration of prognostic nomogram. **(A)** Multivariate analysis of the TCGA cohort. **(B)** Multivariate analysis of the ICGC cohort. **(C)** Multivariate analysis of the GEO cohort. **(D)** Nomogram predicting 3-, 5-, 7- year overall survival. **(E)** Calibration curves. **(F)** Kaplan-Meier curve. **(G)** ROC curves of the nomogram.

Multivariate Cox regression analyses indicated the risk scores were independent factors (*P* < 0.001) in the TCGA-OV (**Figure 10A**), ICGC-OV-AU (**Figure 10B**), and GSE138866 cohorts (**Figure 10C**). Then, a nomogram predicting 3-, 5-, 7-year survival was explored to help clinical practices (**Figure 10D**). Calibration curves indicated that the nomogram had better performance in predicting 5- and 7-year survival (**Figure 10E**).

The KM and ROC curves were also plotted (**Figures 10F, G**).

### Exploration in Clinical Tissues With qRT-PCR

**Figure 11:**
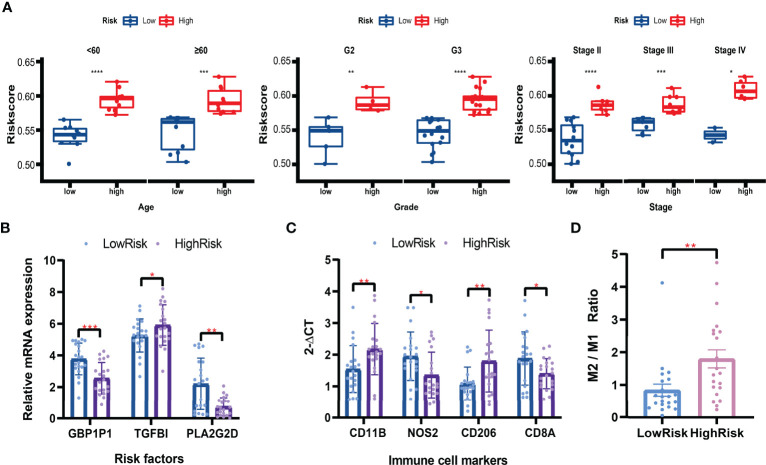
Validation in clinical tissues by qRT-PCR. **(A)** Boxplots of the risk scores in subgroups (Aged ≥ 60, Age < 60, Grade 2, Grade 3, Stage II, Stage III, and Stage IV). Student’s t-test. **(B)** Relative mRNA expression levels (2^-ΔCT^) of risk factors included in the risk model. **(C)** Relative mRNA expression levels (2^-ΔCT^) of immune cell markers. **(D)** The M2/M1 ratio in the high-risk and low-risk patients. Mann-Whitney U test. *P < 0.05; **P < 0.01; ***P < 0.001; ****P < 0.0001.

Based on the formula calculated by the risk score model and the relative mRNA expression levels of risk genes, 42 clinical patients were assigned to the high-low risk group based on the median risk score. In different clinical subgroups (Age ≥ 60, Age < 60, Grade 2, Grade 3, Stage II, Stage III, and Stage IV), the risk scores of the high-risk group of patients were significantly higher than in the low-risk group (**Figure 11A**). The expression levels of GBP1P1 and PLA2G2D were significantly higher in the low-risk group, while the expression level of TGFBI was significantly higher in the high-risk group (**Figure 11B**). Specifically, monocyte marker gene CD11B and M2-type macrophage marker gene CD206 were significantly overexpressed in the high-risk group, while M1-type macrophage marker gene NOS2 was significantly overexpressed in the low-risk group (**Figure 11C**). On the other hand, the CD8T cell marker gene CD8A was significantly overexpressed in the low-risk group (**Figure 11C**). The M2/M1 (CD206/NOS2) ratios in the high-risk patients were significantly higher than those in the low-risk patients (**Figure 11D**). These data suggest that the macrophage polarization and CD8 T cell infiltration might play potentially important roles in the development and prognosis of ovarian cancer.

## Discussion

Immune cell infiltration is a significant feature of tumor cells, regulating the cancer progression and treatment responses (40). For example, B cells play an essential role in the humoral immune response, while T cells participate in cell-mediated immune responses (41, 42). Effective immune responses can eradicate malignant tumor cells or damage their phenotypes and functions. However, cancer cells have evolved various immune evasion mechanisms (43). Despite significant advances represented by secondary cytoreductive surgery and PARP inhibitors that have been taken in the surgical treatments and targeted therapies for OC patients, the benefits generated from immunotherapies remain limited. One of the significant troubles is that only a small population of patients exhibit responses to immunotherapies (44).

Many studies have been devoted to discovering predictors to screen the potential patients who may benefit from immunotherapy. Patients with phenotypes like PD-L1 positive (45), high microsatellite instability (MSI-H), different Mismatch Repair (dMMR) (46), high Tumor Mutational Burden (TMB), T-cell-inflamed Gene Expression Profiles (GEP) have significantly higher immune response rates in multiple cancers (47). Increasing applications of next-generation sequencing and other genome technologies reveal the heterogeneity in genetic and molecular levels among cancer patients (48). The genomics data explosion also promotes the development of various algorithms to learn big data deeply.

Ovarian cancer is also a heterogeneous disease in which the differences in the immune microenvironment have received more and more attention (49). Immunotyping plays an essential role in the immunotherapy and prognosis of patients (50). In our study, 75 characteristic genes that led to the heterogeneity in ICI were identified, and three ICI-related immunotypes with significant differences in prognosis were defined. Among the 22 immune cell types, macrophages and T cells had a higher degree of infiltration, and the differences among the three immunotypes were the greatest. Tumor-associated macrophages (TAMs) are the main immune cells in the ovarian tumor microenvironment (51). It is already known that M1 macrophages inhibit tumor progression while M2 has the opposite effect (52). Also, the expressions of multiple immune checkpoint-related immunotherapy target genes vary significantly among the three immunotypes, providing guidance for immunotherapy strategies in different patients (53). In detail, PD-1, CTLA-4, LAG3, and TIGIT immune checkpoint blockade therapies might be more effective to ICI cluster A. B7-H3 blockade therapy might be better for ICI cluster B, and TIM-3 might be better for ICI cluster C. CD8 TCR pathway, chemokine-mediated signaling pathway, B cell receptor signaling pathway and adaptive immune response were revealed to be the mainly enriched pathways or biological functions for the immunophenotyping.

Based on the 75 immunophenotyping-related characteristic genes, we explored a novel biomarker, the ICI score. Patients with ICI scores over -2.946049 were defined as the high-ICI group, which showed a better prognosis. Patients with higher TMB values over 1.315789 were defined as the high-TMB group, with a better prognosis too. The combination of the ICI score and TMB exhibited higher resolution to prognostic immunotypes. Specifically, patients with the ICI score > -2.946049 & TMB > 1.315789 had the best overall survival, and patients with the ICI score < -2.946049 & TMB < 1.315789 had the worst, while patients with the ICI score < -2.946049 & TMB > 1.315789 or the ICI score > -2.946049 & TMB < 1.315789 had similar and moderate prognosis.

However, the higher number of model genes corresponds to the higher cost and difficulty of clinical application. Finally, we explored another independent prognostic biomarker, the risk score, to distinguish high-risk and low-risk patients. The risk score could be calculated based on the three genes’ mRNA expressions. Patients with risk scores > 1.034349 had worse prognoses, more serious tumor immune dysfunction and exclusion, lower immunogenicity, and lower immunotherapy responses. Meanwhile, the fractions of antitumor immune cells like T cells CD8, NK cells activated, Macrophages M1 were significantly higher in low-risk patients, consistent with the ICI clusters results.

Our study systematically revealed the characteristics of immune cell infiltration in ovarian cancer patients, screened out characteristic genes associated with immune typing, and integrated novel prognostic markers which could predict patients’ immunotherapy responses. Compared with similar published articles, the expression data of the three independent cohorts included in our study are all generated from next-generation sequencing technology. However, the cohorts included in many reports were mixed from two different technology platforms, gene chip and next-generation sequencing, which significantly reduced the reliability of the results (54). For example, several published articles in a similar direction have combined chip and sequencing datasets using Combat algorithm (55, 56). However, according to recent literature, there are still some differences in data distribution after using Combat to remove the batch effect between these two different technology platforms (57). On the other hand, there are 117 genes included in the ICI score model in Liu’s research (55) and 274 genes in Li’s study (56). In contrast, our study’s ICI score based on 75 characteristic genes has a smaller number of genes and is more conducive to clinical application. Moreover, our research made more efforts to develop a more streamlined, efficient, and robust model. The model comparison results also show that our 3-gene ICI signature contains the least number of variables and is at the forefront of accuracy compared with the recently published models. In addition, we confirmed significant results of immune infiltration in clinical tissues and observed differential expression of risk genes and macrophage polarization and CD8 T cell marker genes.

There are also limitations in the present study. First, the sample size is not large enough, and the stability of immunophenotyping needs to be verified in a larger sample size cohort. Second, the number of characteristic genes related to immune typing is large, and further simplification will be helpful for clinical application. Thirdly, further studies on the molecular mechanism behind the immunotyping will benefit the development of immunotherapy strategies.

## Conclusions

This study characterized the landscape of the immunotypes and provided novel immune infiltration-related prognostic biomarkers in ovarian cancer, guiding the survival prediction and immunotherapy strategies in the clinic.

## Data Availability Statement

The original contributions presented in the study are included in the article/**Supplementary Material**. Further inquiries can be directed to the corresponding authors.

## Ethics Statement

The studies involving human participants were reviewed and approved by the Ethics Committee of Dongying People’s Hospital. The patients/participants provided their written informed consent to participate in this study.

## Author Contributions

NZ, YX and HC analyzed and interpreted the data. HC and YH designed the study and were major contributors in writing the manuscript. All authors read and approved the final manuscript. All authors contributed to the article and approved the submitted version.

## Conflict of Interest

The authors declare that the research was conducted in the absence of any commercial or financial relationships that could be construed as a potential conflict of interest.

## Publisher’s Note

All claims expressed in this article are solely those of the authors and do not necessarily represent those of their affiliated organizations, or those of the publisher, the editors and the reviewers. Any product that may be evaluated in this article, or claim that may be made by its manufacturer, is not guaranteed or endorsed by the publisher.

## Supplementary Material

Supplementary Figure 1 Violin plots of four immune checkpoint genes. Wilcoxon test. ns: not significant.

Supplementary Figure 2 PCA analyses before and after removing the batch effects.

Supplementary File S1 The immune cell infiltrations calculated in the TCGA cohort with CIBERSORT.

Supplementary File S2 Immune and stromal scores calculated with ESTIMATE.

Supplementary File S3 The list of the DEGs and characteristic genes screened with Boruta.

Supplementary File S4 ICI scores of all the patients included in this study.

Supplementary File S5 TMB values of the TCGA-OV cohort.

Supplementary File S6 Univariate Cox regression analysis result.

Supplementary File S7 Risk scores and clinical data in the IMvigor210 cohort.

Supplementary File S8 Risk scores and IPS data in TCIA database.
