# Supplementary figures and images for: Exploration of the Immunotyping Landscape and Immune Infiltration-Related Prognostic Markers in Ovarian Cancer Patients
Source: Front Oncol. 2022 Jul 8;12:916251. doi: 10.3389/fonc.2022.916251 (PMC9307664; doi:10.3389/fonc.2022.916251)

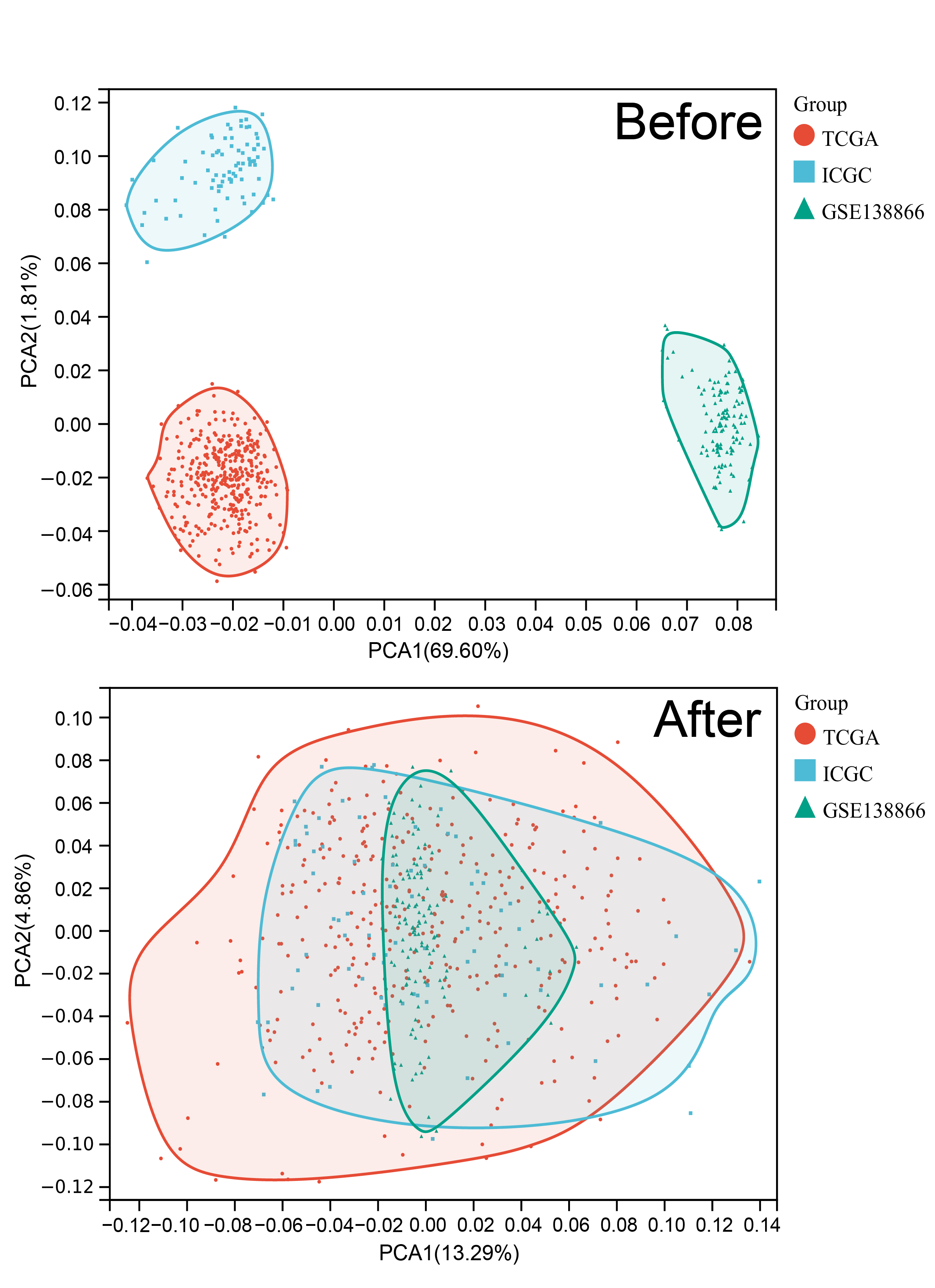

Supplement: Supplementary Figure 1 — Violin plots of four immune checkpoint genes. Wilcoxon test. ns: not significant. [file Image_1.tif]

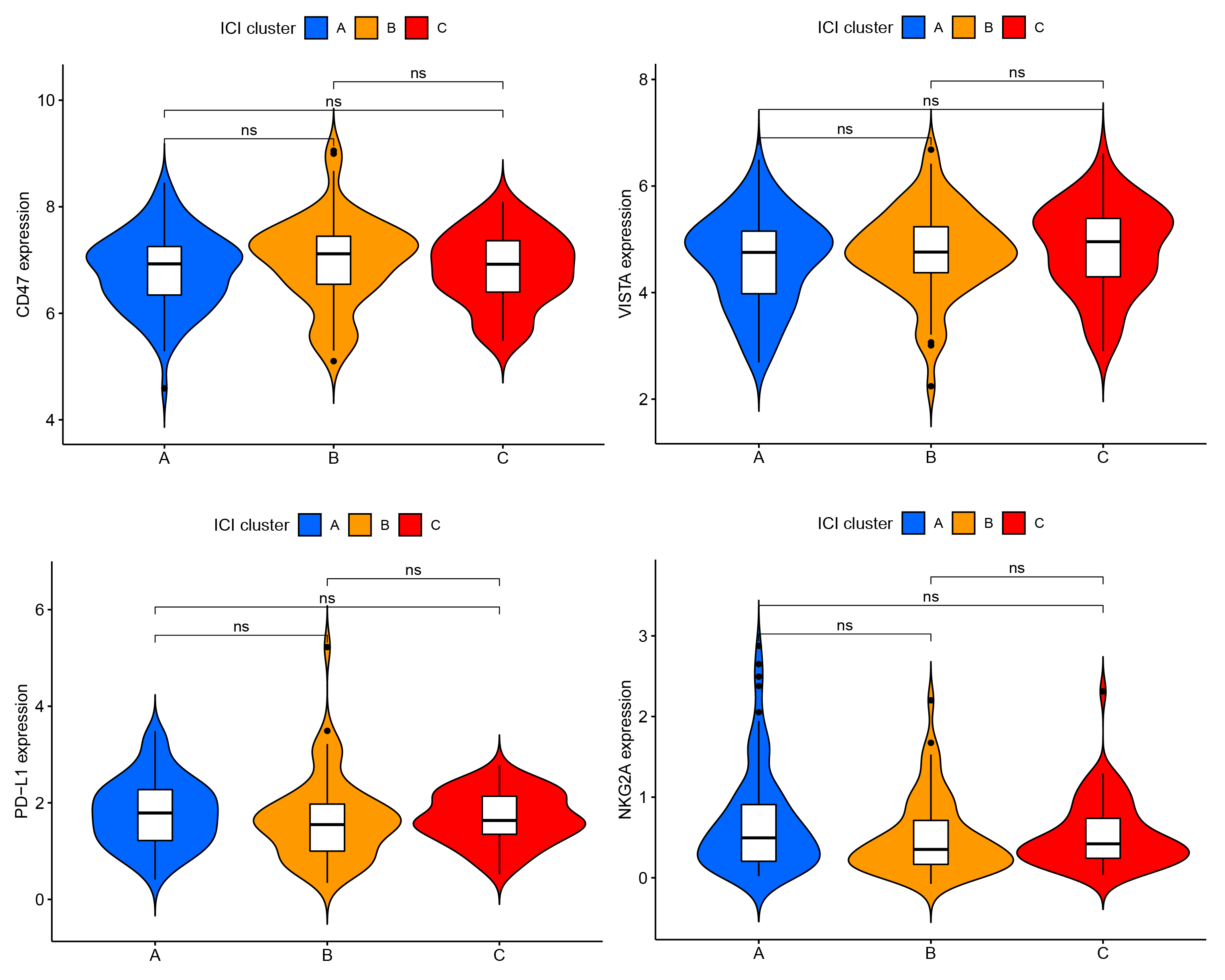

Supplement: Supplementary Figure 2 — PCA analyses before and after removing the batch effects. [file Image_2.tif]
